# A Case of Squamous Cell Carcinoma Arising From an Intraductal Papilloma of the Breast

**DOI:** 10.1002/ccr3.71068

**Published:** 2025-09-28

**Authors:** Kanako Nishiyama, Kumiko Okujima, Yosuke Mizuno

**Affiliations:** ^1^ Department of Breast Surgery Matsuyama Red Cross Hospital Matsuyama Japan; ^2^ Department of Pathology Matsuyama Red Cross Hospital Matsuyama Japan

**Keywords:** breast cancer, intraductal papilloma, squamous cell carcinoma, squamous differentiation

## Abstract

We present a rare case of squamous cell carcinoma arising from an intraductal papilloma in a 60‐year‐old female. Histopathology revealed an invasive squamous cell carcinoma with p63 positivity, coexisting with a benign papilloma. Following partial mastectomy and adjuvant therapy, the patient remained disease‐free for 4 years and 8 months, highlighting the importance of early detection.

## Graphical abstract

Graphical abstract showing squamous cell carcinoma arising from an intraductal papilloma of the breast.

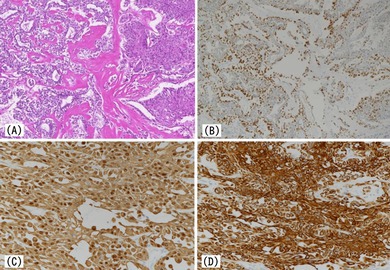

**Summary**

- This rare case highlights invasive squamous cell carcinoma arising from an intraductal papilloma, emphasizing early detection and aggressive re‐biopsy for accurate diagnosis and effective treatment.

## 1 Introduction

Intraductal papilloma (IDP) is a benign breast tumor arising from the mammary duct epithelium, with an incidence of 2%–3% [1]. Diagnosis typically involves imaging followed by histological confirmation to rule out malignancy. IDPs are known to undergo metaplastic changes (apocrine, squamous, and sebaceous) as a reactive process [2]. Squamous metaplasia in papilloma is rare, usually benign, and typically presents as a small focus in all cases. In extreme cases, papillomas can undergo complete squamous metaplasia and transform into squamous cell carcinoma (SqCC) in situ [3, 4]. Here, we report a rare case of a patient with an IDP who underwent complete squamous metaplasia and malignant transformation.

## 2 Case History/Examination

**FIGURE 1:**
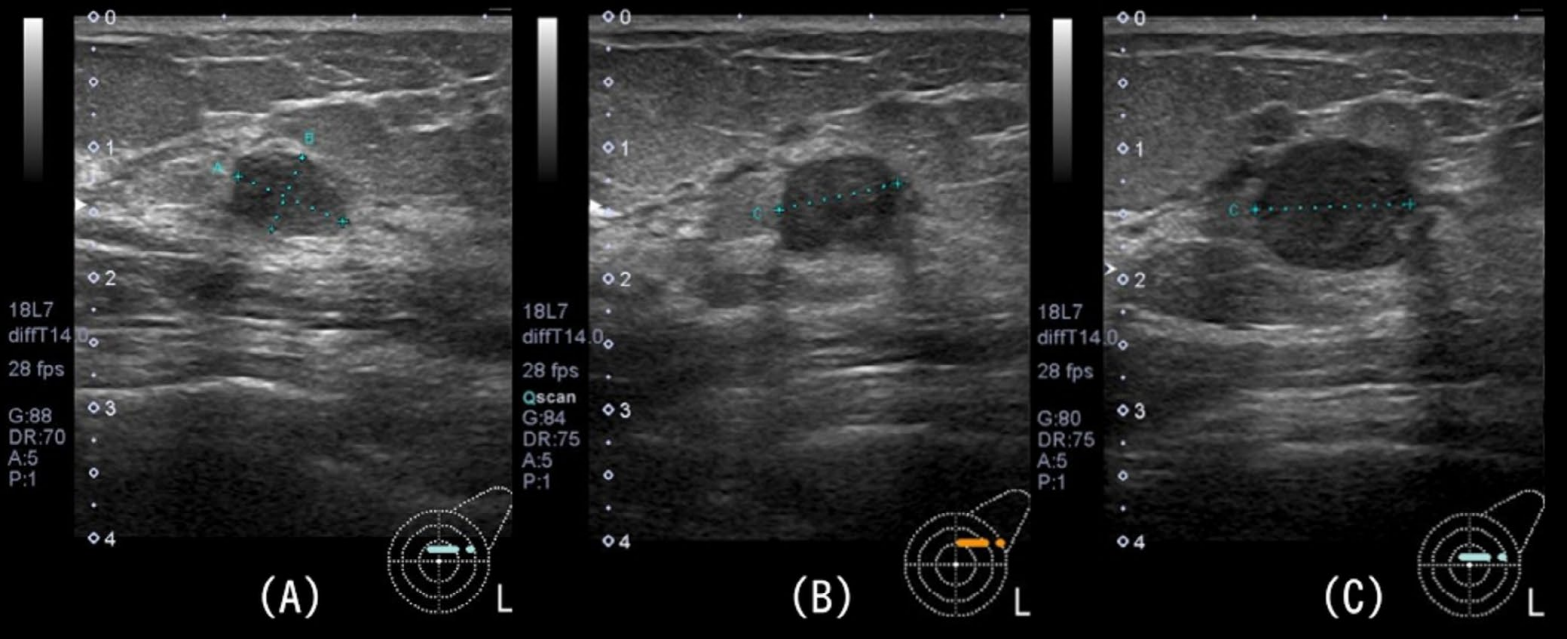
Serial ultrasound images of the left breast mass. (A) Initial presentation: The lesion was identified and diagnosed as an IDP via needle biopsy. (B) Six months later: A slight increase in size was observed. (C) One year later: The lesion demonstrated further enlargement with irregular margins, raising suspicion of malignancy.

A 60‐year‐old female with no significant family history was referred to our department 4 years earlier following an abnormal breast cancer screening result. Imaging revealed benign findings in the right breast, which remained stable without notable changes during follow‐up. However, routine follow‐up revealed a newly developed mass in the left breast. Mammography identified a focal asymmetrical density (FAD) in the upper outer quadrant of the left breast. Breast ultrasonography further showed a well‐defined, smooth, hypoechoic mass measuring 9.2 × 8.8 × 6.0 mm (Figure 1A). A core needle biopsy was performed, and the lesion was diagnosed as benign IDP.

Six months later, follow‐up ultrasonography revealed an increase in tumor size to approximately 11.2 mm (Figure 1B). Another 6 months later, the lesion further enlarged to 13 mm (Figure 1C), prompting a repeat vacuum‐assisted biopsy. Histopathological examination revealed the coexistence of benign IDP and invasive carcinoma with squamous differentiation. Immunohistochemical analysis showed that the tumor was negative for the estrogen receptor (ER), progesterone receptor (PgR), and HER2, confirming the diagnosis of triple‐negative breast cancer (TNBC).

## 3 Differential Diagnosis

**FIGURE 2:**
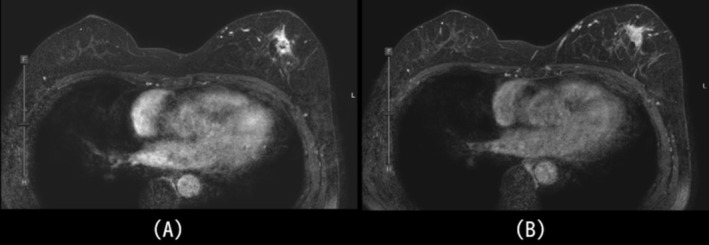
Dynamic contrast‐enhanced MRI images of the breast. (A) Early‐phase image shows a 1.3‐cm irregular mass with rapid enhancement. (B) Delayed‐phase image demonstrates persistent enhancement with irregular margins.

After confirmation of SqCC on biopsy, contrast‐enhanced MRI was performed to evaluate the extent of disease and assist in surgical planning. Contrast‐enhanced MRI demonstrated a 1.1‐cm mass in the upper outer quadrant of the left breast, showing heterogeneous internal fast‐washout enhancement and surrounding regional non‐mass enhancement (Figure 2). Based on these findings, we considered primary breast cancer, particularly invasive ductal carcinoma arising from ductal carcinoma in situ (DCIS), or IDP with squamous metaplasia for the differential diagnosis. Re‐biopsy and histopathology confirmed invasive SqCC arising from pre‐existing IDP.

## 4 Conclusion and Results

**FIGURE 3:**
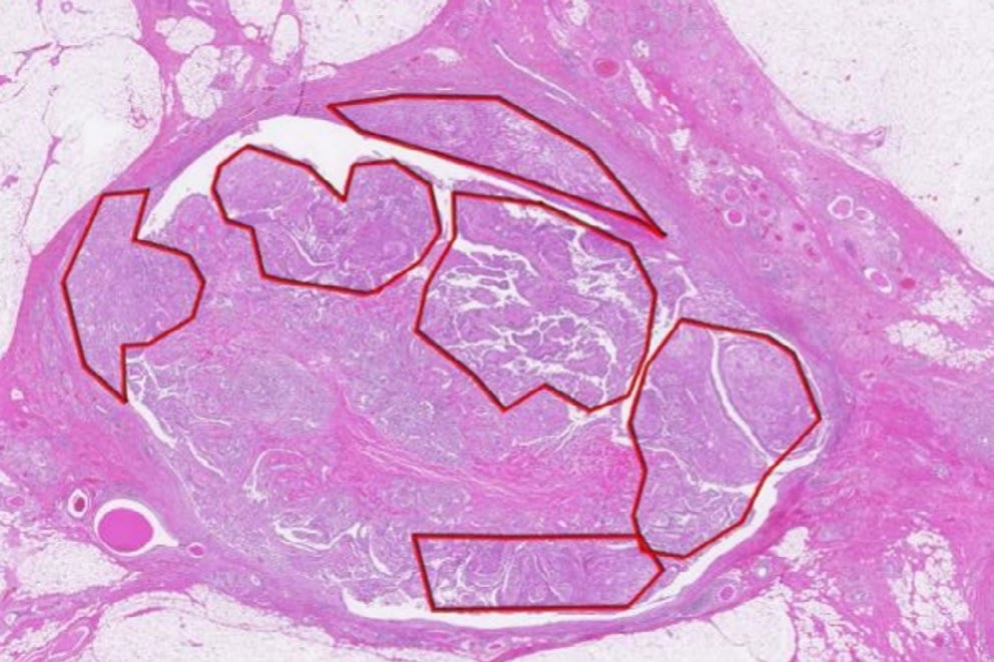
Low‐power view of the left breast tumor. Hematoxylin and eosin staining revealed SqCC components (outlined in red) intermingled with areas of benign IDP. In some regions, SqCC infiltrated beyond the ductal structures.

**FIGURE 4:**
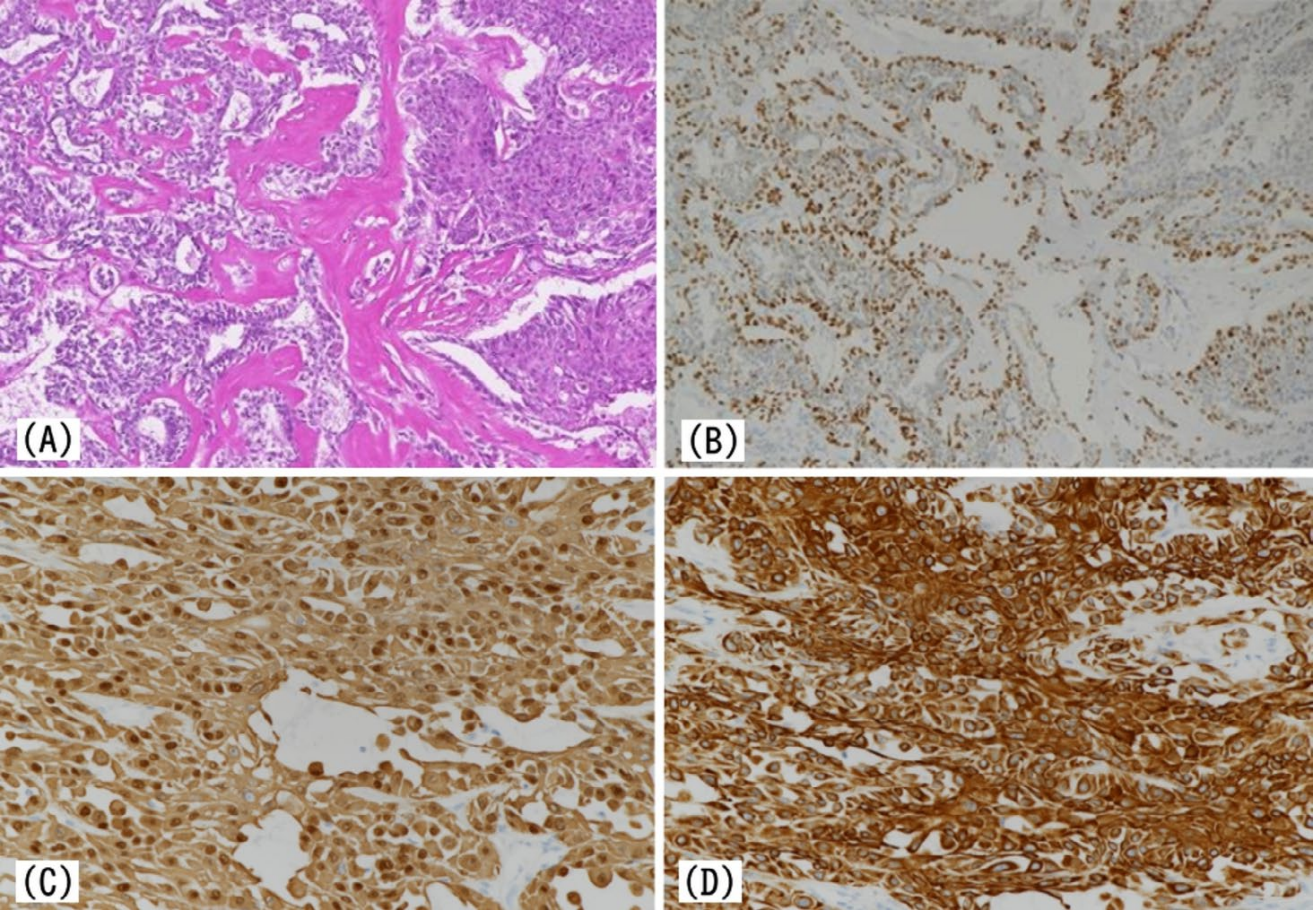
Histological and immunohistochemical findings of the left breast tumor. (A, ×100) Hematoxylin and eosin staining shows adjacent components of intraductal papilloma (left) and squamous cell carcinoma (right). (B, ×100) p63 immunostaining reveals nuclear positivity in the SqCC component. (C, ×200) Dual staining with a p40 and CK14 cocktail shows cytoplasmic CK14 expression and focal nuclear positivity for p40 in tumor cells. (D, ×200) CK5/6 immunostaining demonstrates diffuse cytoplasmic positivity.

Based on these findings, the patient was diagnosed with left breast cancer, cT1N0M0, Stage I. Surgical management involved a partial mastectomy with sentinel lymph node biopsy. No lymph node metastasis was detected, and axillary dissection was omitted. Histopathological examination after surgery confirmed invasive SqCC with a maximum invasion diameter of 10 mm coexisting with areas of benign IDP (Figure 3). Immunohistochemical analysis demonstrated p63 positivity in the tumor cells (Figure 4), consistent with squamous differentiation. Additional immunostaining revealed focal nuclear positivity for p40, with diffuse cytoplasmic positivity for CK5/6 and CK14, further supporting squamous differentiation. Notably, the lesion exhibited biphasic characteristics comprising areas of benign IDP and atypical squamous epithelial cells infiltrating the stroma, with no evidence of glandular carcinoma components. Based on these findings, the tumor was diagnosed as SqCC arising from pre‐existing IDP.

The patient underwent adjuvant chemotherapy and radiotherapy. At 4 years and 8 months postoperatively, there was no evidence of recurrence and the patient remained disease‐free.

## 5 Discussion

Primary SqCC of the breast is a rare malignancy, accounting for less than 0.1% of all invasive breast cancers [5, 6]. It is thought to arise from the squamous metaplasia of ductal carcinoma cells or the mammary ductal epithelium. Breast SqCC tumors are generally larger than conventional breast cancer histological subtypes, with an average size exceeding 5 cm. Breast SqCC typically occurs in elderly patients and is associated with a low incidence of lymph node metastases but carries a risk of distant metastases [5, 6]. These tumors lack estrogen and progesterone receptors and are known to exhibit resistance to conventional breast cancer chemotherapy regimens [7]. Importantly, the diagnosis of breast SqCC requires the exclusion of other primary sites of SqCC within the body. Although treatment often follows standard breast cancer protocols, including mastectomy and lymph node dissection, the prognosis remains unclear and no definitive treatment guidelines for breast SqCC have been established [7].

Although SqCC is typically identified as an advanced invasive carcinoma, non‐invasive SqCC of the breast is an extremely rare entity characterized by the presence of pure squamous cells without glandular differentiation [4]. In contrast, DCIS is defined by the presence of malignant epithelial cells confined within the ducts, without invasion beyond the basement membrane. DCIS exhibits various structural patterns, including solid, cribriform, micropapillary, and mixed types [8]. In situ squamous cell carcinoma is exceptionally rare, with only a few reported cases in the literature [4, 9, 10]. Histologically, these cases exhibit distinctive features such as keratinized squamous cells, intercellular bridges, and keratin pearl formation. Immunohistochemical analyses typically demonstrate positivity for markers associated with squamous differentiation, including p63, p40, and cytokeratin.

A summary of the clinicopathological features of previously reported cases, along with the present case, is provided in Table 1. These tumors were generally detected at an early stage with small tumor size, and outcomes were favorable following surgical resection and adjuvant radiotherapy or chemotherapy. While squamous differentiation is a common hallmark, evidenced by keratinization, intercellular bridges, and keratin pearls, one report by Hayes et al. [10] has demonstrated additional immunohistochemical features indicative of both myoepithelial and luminal cell differentiation. These findings suggest that SqCC in situ may not represent a uniform entity but rather a lesion with multilineage differentiation potential, implying the involvement of progenitor cells with broader epithelial plasticity at the in situ stage of breast cancer development.

**TABLE 1:** Reported cases of squamous cell carcinoma in situ of the breast.

Case	Age	Presentation	Extent (cm)	Architecture	Immuno findings	Treatment	Follow‐up
Hayes et al. (2007) Case 1	59	Screening mammography	0.5	SqCC in situ	p63+, CK14+, Actin+(focal), myosin+(focal), EGFR+, triple negative	Wide excision + RT	18 months, NED
Hayes et al. (2007) Case 2	35	Palpable mass	2.0	SqCC in situ	p63 + (focal), CK14 (patchy), actin+, CK7+, EMA+, p53+ triple negative	Simple mastectomy + RT	15 months, NED
Hayes et al. (2007) Case 3	51	Screening mammography	1.8	SqCC in situ + invasive component	p63+, CK14 + (patchy), CK7+, EMA+(focal), EGFR+(focal) triple negative	Wide excision + RT	10 months, NED
Arafah et al. (2016)	73	Screening mammography	1.6	SqCC in situ	P63+, p40+, triple negative	Simple mastectomy	132 months, NED
Lu et al. (2023)	85	Screening mammography	1.6	SqCC in situ	p63+, p40+, CK5/6+, triple negative	Simple mastectomy	38 months, NED
Mayorga et al. (2023)	64	Palpable painful mass	2.0	SqCC in situ with IDP	Collagen IV+ for in situ, other IHC not reported	Excisional biopsy	LTFU
Present case (2025)	60	Follow‐up imaging, progressive growth	1.3	SqCC in situ + invasive component, with IDP	p63+, p40 + (patchy), CK14+, CK5/6+, triple negative	Partial mastectomy + RT + CT	56 months, NED

Abbreviations: CT, chemotherapy; LTFU, lost to follow‐up; NED, no evidence of disease; RT, radiotherapy; SqCC, squamous cell carcinoma.

Squamous metaplasia of the breast is an exceptionally rare pathology that can closely resemble malignant lesions on imaging and fine needle aspiration biopsy [11]. Chronic inflammation, particularly in response to foreign materials such as silicone implants, has been identified as a potential causative factor and may play a pivotal role in the development of squamous metaplasia [12]. This phenomenon has been observed in various breast lesions, including IDP and fibroadenoma, and often raises a strong suspicion of malignancy. To minimize unnecessary surgical intervention, intraoperative frozen section analysis has been demonstrated as a critical tool for distinguishing benign from malignant lesions [11].

In our case, the lesion was initially diagnosed as a benign intraductal papilloma on core needle biopsy and was followed with serial imaging. However, progressive enlargement and the development of irregular margins raised suspicion for malignancy, prompting repeat biopsy and surgical resection. This highlights the importance of continued vigilance in managing intraductal lesions, even those initially considered benign. In particular, when subtle but progressive changes are noted on imaging, such as an increase in size, margin irregularity, or evolving internal architecture, prompt re‐evaluation is warranted. While squamous cell carcinoma of the breast is generally associated with a poor prognosis in its invasive form, curative treatment is achievable when the disease is identified and managed at the in situ stage. Therefore, accurate assessment of progressive imaging changes in seemingly benign lesions is critical to ensuring timely diagnosis and optimal outcomes.

## Author Contributions

**Kanako Nishiyama:** conceptualization, data curation, investigation, writing – original draft, writing – review and editing. **Kumiko Okujima:** writing – review and editing. **Yosuke Mizuno:** data curation, investigation.

## Consent

Written informed consent was obtained from the patient for the publication of this case report and any accompanying images.

## Conflicts of Interest

The authors declare no conflicts of interest.

## Data Availability

Data sharing is not applicable to this article as no datasets were generated or analyzed during the current study.
